# Constraints on fundamental physical constants from bio-friendly viscosity and diffusion

**DOI:** 10.1126/sciadv.adh9024

**Published:** 2023-08-23

**Authors:** Kostya Trachenko

**Affiliations:** School of Physical and Chemical Sciences, Queen Mary University of London, Mile End Road, London E1 4NS, UK.

## Abstract

The problem of understanding fundamental physical constants and their values was discussed in particle physics, astronomy, and cosmology. Here, I show that an additional unexpected insight comes from condensed matter physics and liquid physics in particular: Fundamental constants have a biofriendly window constrained by biofriendly viscosity and diffusion setting the motion in essential life processes in and across cells. I also show that bounds on viscosity, diffusion, and the fundamental velocity gradient in a biochemical machine can all be varied while keeping the fine-structure constant and the proton-to-electron mass ratio intact, with no implication for the production of heavy nuclei in stars. This leads to a conjecture of multiple tuning and an evolutionary mechanism.

## Teaser

Liquid viscosity imposes unexpected extra constraints on fundamental physical constants.

## INTRODUCTION

The origin and values of fundamental physical constants were discussed in high-energy particle physics, cosmology, and astronomy (*1*–*10*). These constants give our world its distinctive character and differentiate it from others we might imagine. The values of these constants have no explanation and are therefore considered arbitrary (*4*), for the reason that we do not know what kind of theories we need to explain them (*10*). Understanding fundamental constants is viewed as one of the grandest questions in modern science (*11*).

The values of some fundamental physical constants are considered to be finely tuned and balanced to give our observable world. Examples include finely tuned balance between quark masses needed to produce protons and neutrons (*5*, *6*, *12*) and production of heavy nuclei in stars, which depends on the finely tuned balance between the fine structure constant $\alpha =\frac{e^{2}}{\hbar c}\approx \frac{1}{137}$ (*e* is the electron charge and *c* is the speed of light) and the ratio of the proton mass *m_p_* and electron mass *m_e_*, $\beta =\frac{m_{p}}{m_{e}}\approx 1836$. These and other example suggest a narrow “habitable zone” in parameter space (α,β) where essential biochemical elements can form (*1*, *8*, *9*) [see, however, (*6*)]. For this reason, fundamental constants are referred to as “biofriendly” or “biophilic” (*1*, *6*). Trying to rationalize fundamental constants, their balance and tuning has given rise to the anthropic principle (*1*–*3*, *5*–*9*).

Discussions of constraints on fundamental constants and their fine-tuning involve high-energy processes at different scales and often end with production of heavy nuclei in stars. This involves a tacit assumption that once heavy nuclei are produced, observers emerge. However, there are about 15 orders of magnitude size difference between nuclei and observers. Many life processes, including the formation of proteins, RNA, living cells, and so on, operate on the scale of length and energy considered in condensed matter physics. Because of their complexity and variety, these processes were not thought to be describable by a physical model, which can relate them to fundamental constants and put biofriendly constraints on these constants (*2*, *13*). The challenge is to have a physical model, which is both general enough to be widely applicable and specific enough to connect life processes directly to fundamental constants.

Here, I show that these models are nevertheless possible. These models are general enough to impose constraints on fundamental constants from biofriendly viscosity and diffusion involved in essential life processes setting the motion in and across cells. These constraints imply a biofriendly window for fundamental constants. I show that bounds on viscosity and diffusion can be varied while keeping α and β intact, with no implication for the production of heavy nuclei. The same applies to the fundamental velocity gradient that I introduce in relation to flow in a biochemical machine. These observations lead to a conjecture of multiple tuning and an evolutionary mechanism.

## RESULTS AND DISCUSSION

### Motion and flow

I consider the cell, the basic building block of life forms. There are several areas related to cells where flow is important. The two important ones are the operation of the cell itself [e.g., transport involving protein motors and cytoskeletal filaments, passive and active molecular transport, cytoplasmic mixing, mobility of cytoplasmic constituents, diffusion involved in cell proliferation (*14*, *15*), and so on] and the flow in the organism involving many cells (e.g., blood flow). Another area where flow is important is related to the prebiotic synthesis of life building blocks in the metabolic flux, the basis of life, thought to give rise to DNA blocks in protocells (*16*). Liquids and gases are two states providing a medium where this flow can happen and matter can move. Viscosity governs this flow and is therefore tightly embedded in life processes and their dynamics.

In our world, the motion-enabling liquid is water; however, the physical model discussed below and its implications apply to all liquids. If life in a different world is not water-based but uses another liquid as a medium to provide motion, the model implications are the same.

**Fig. 1.**
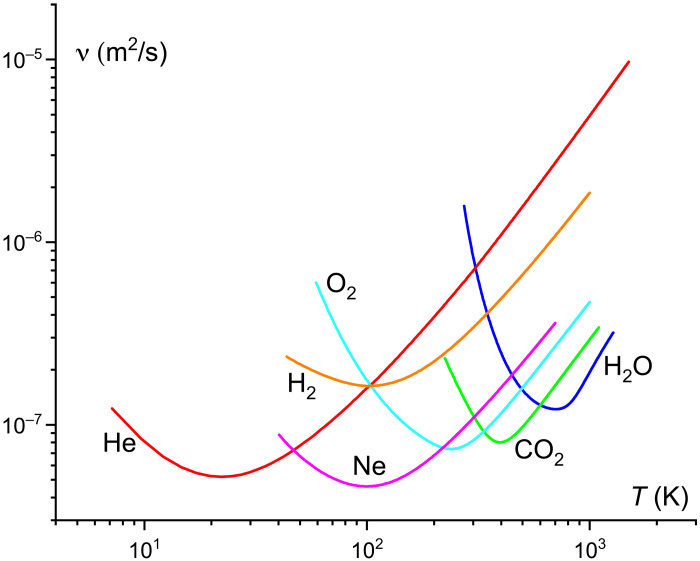
Experimental kinematic viscosity of noble, molecular, and network fluids showing minima. ν for He, H_2_, O_2_, Ne, CO_2_, and H_2_O are shown at 20, 50, 30, 50, 30, and 100 MPa, respectively. The experimental data are from (*26*).

I recall that viscosity, universally, has minima seen in Fig. 1. The minima correspond to the crossover between liquid-like and gas-like dynamics. The data in Fig. 1 are shown above the supercritical pressure to extend the temperature range where the system is a fluid. Above the critical point, the minima are smooth and slightly increase with pressure. Below the critical point, viscosity has sharper change at the liquid-gas transition; however, viscosity minima above and below the critical point are close (*17*).

The kinematic viscosity ν at the minimum, ν_min_, can be evaluated as

$$\nu_{\min}=\frac{1}{4\pi }\frac{\hbar }{\sqrt{m_{e}m}}$$ (1)

where *m* is the molecule mass and is in agreement with viscosity minima seen in a wide range of experimental data (*17*). The lower viscosity bound (Eq. 1) is also consistent with the high-temperature limit of the experimental viscosity of metallic liquids (*18*).

I now ask what constraints are imposed on the fundamental constants from essential life processes in and between cells where motion and flow are involved. Let us write the Navier-Stokes equation as

$$\rho \frac{\partial v}{\partial t}=-p+\eta \nabla^{2}v$$ (2)

where *v* is the fluid velocity, which is assumed to be small; *p* is the pressure; ρ is the density; and η is the dynamic viscosity.

For time-dependent nonequilibrium flow, the solution of Eq. 2 depends on kinematic viscosity $\nu =\frac{\eta }{\rho }$. For steady flow, flow velocity depends on η. The minimum of η, η_min_, can be evaluated as η_min_ = ν_min_ρ, where density $\rho \propto \frac{m}{a_{B}^{3}}$ and *a*_B_ is the Bohr radius

$$a_{B}=\frac{4\pi \varepsilon_{0}\hbar^{2}}{m_{e}e^{2}}$$ (3)

where *e* and *m_e_* are electron charge and mass, respectively.

Assuming that *m* = *Am_p_*, where *A* is atomic number and setting *A* = 1 for the purpose of the following discussion, this gives

$$\eta_{\min}\propto \frac{e^{6}}{\hbar^{5}}\sqrt{m_{p}m_{e}^{5}}$$ (4)

A useful property related to viscosity is the liquid relaxation time, the average time it takes a molecule to jump from one quasi-equilibrium place to the next. Its minimal value, τ_min_, gives a characteristic “elementary” time associated with molecular motion. τ_min_ can be evaluated using the Maxwell relation as $\tau_{\min}=\frac{\eta_{\min}}{G}$ (*19*), where *G* is the high-frequency shear modulus. Using Eq. 4, recalling that the upper bound of elastic moduli is set by fundamental constants as $G\propto \frac{E_{R}}{a_{B}^{3}}$ (*20*), where *E*_R_ is the Rydberg energy

$$E_{R}=\frac{m_{e}e^{4}}{32\pi^{2}\varepsilon_{0}^{2}\hbar^{2}}$$ (5)

gives

$$\tau_{\min}\propto \frac{\hbar^{3}}{m_{e}e^{4}}{\left(\frac{m_{p}}{m_{e}}\right)}^{\frac{1}{2}}$$ (6)

τ_min_ is related to the shortest time scale in the system set by the Debye vibration period, τ_D_. Writing $\tau_{D}=\frac{1}{\omega_{D}}$, where ω_D_ is Debye frequency, recalling $\hbar \omega_{D}=E{\left(\frac{m_{e}}{m}\right)}^{\frac{1}{2}}$ (*17*), where *E* is cohesive energy, setting *a* and *E* to their characteristic scales *a*_B_ and *E*_R_ and using *m* = *Am_p_* with *A* = 1 as before gives τ_min_ = τ_D_ up to a constant.

As compared to η_min_ in Eq. 4, τ_min_ depends on fundamental constants differently: For example, smaller ℏ increases η_min_ but decreases τ_min_. Physically, this is because smaller ℏ gives larger *E*_R_, increasing the bond energy and bond stiffness. This increases ω_D_ and decreases τ_D_. As a result, τ_min_ ∝ τ_D_ decreases. Hence, τ_min_, which sets the time scale of short-time dynamics, becomes faster in response to the variation of fundamental constants, which increases *E*_R_. On the other hand, viscosity and its minimal value η_min_ increase with *E*_R_ and with the variation of fundamental constants causing this increase: For example, smaller ℏ increases both *E*_R_ in Eq. 5 and η_min_ in Eq. 4. I will revisit this point below.

η_min_ in Eq. 4 corresponds to maximal diffusion constant *D* as follows from the Stokes-Einstein relation $D=\frac{k_{B}T}{6\pi r\eta }$, where *r* is the radius of a moving particle. This gives

$$D_{\max}\propto \frac{1}{\eta_{m}}\propto \frac{\hbar^{5}}{e^{6}}\frac{1}{\sqrt{m_{p}m_{e}^{5}}}$$ (7)

Eqs. 1, 4, 6, and 7 set the limits for important properties governing dynamics, motion, and flow in terms of fundamental physical constants. I now flip the question and ask what happens to these properties if the fundamental constants were different?

η_min_ in Eq. 4 and D_max_ in Eq. 7 are quite sensitive to ℏ and *e*. If the viscosity minimum η_min_ increases due to, for example, smaller ℏ or larger *e*, then viscosity necessarily becomes higher at all conditions of pressure and temperature, in all liquids (and not just in water essential in our world). This is seen in Fig. 1. At the same time, diffusion decreases according to Eq. 7, slowing down all diffusive processes of essential substances in and across cells. Physically, the origin of this slowing down due to smaller ℏ or larger *e* is related to the decrease of the Bohr radius (Eq. 3). This results in the increase of the cohesive energy $E=\frac{\hbar^{2}}{2m_{e}a_{B}^{2}}$, which is the Rydberg energy (Eq. 5). The increase of cohesive energy makes it harder to flow and diffuse because a flow event requires overcoming the energy barrier set by the cohesive energy.

Higher viscosity means that water flows slower, notably affecting vital flow processes in and between cells and so on. Large viscosity increase (think of viscosity of tar and higher) means that life might not exist in its current form or not exist at all. Consider, for example, blood viscosity, its normal range is about (3.5 to 5.5) cP. Were viscosity to move substantially outside this range, body functions would be disabled. Changing ℏ or *e* in Eq. 4 by a few percent only already covers the normal range and precludes substantially larger variations of these constants. Higher viscosity also slows down essential chemical reactions involved in life processes such proteins folding and enzyme kinetics (*21*–*23*).

One might ask if viscosity increase due to different fundamental constants may be part of the overall slowing down (similar to a video slow motion), whereby all processes slow down but remain functioning. Several observations can be made in this regard. First, larger viscosity not only slows down dynamics but can arrest a life process. Examples include a transition corresponding to the explosive increase of the coagulation rate in biological fluids such as protein solutions and blood. This takes place at the critical value of the Péclet number, which depends on viscosity (*24*). Second, η_min_ in Eq. 4 increases with *e* and *m_e_* and decreases with ℏ, whereas the elementary time τ_min_ in Eq. 6 or the shortest time τ_D_ do the opposite. As η_min_ and viscosity increase due to, for example, larger *e* or *m_e_* or smaller ℏ, τ_min_ decreases. This implies that in terms of the shortest atomic time scale τ_min_ (τ_D_), time effectively runs faster and processes dependent on short-time dynamics speed up rather than slow down. Third, the chemical reaction rates of vital biological processes involving, for example, dynamics of proteins and enzymes, *k*, vary as $k\propto \frac{1}{\eta^{n}}$, where *n* varies in quite a large range from 0.3 (*21*) to 2.4 depending on the reaction [see, e.g., (*23*) for review]. Therefore, viscosity increase affects different reaction rates differently and disrupts the existing balance between products of different reactions and important interactions between those products. Depending on the degree and nature of this disruption, the result can either be finding a new functioning sustainable balance during life development and hence a different type of life or not finding a sustainable living state at all.

One might also ask if cellular life could find a hotter place where overly-viscous and biounfriendly water is thinned. This would not work: η_min_ and ν_min_ set the minimum below which viscosity cannot fall regardless of temperature or pressure (see Fig. 1). This applies to any liquid and not just water and therefore to all life forms using the liquid state to function.

### Biofriendly window

Let η_0_ and ν_0_ be viscosities above which life processes are disabled and *D*_0_ be the diffusion constant below which life processes are disabled. Conditions for viscosity and diffusion to be biofriendly are

$$\begin{array}{c} \eta_{\min}<\eta_{0}\\ \nu_{\min}<\nu_{0}\\ D_{\max}>D_{0} \end{array}$$ (8)

Each property, η, ν, and *D* acts in different life processes and can therefore disable them independently. η sets steady flow under external pressure gradient such as active transport or flow in a biochemical machine discussed below. For time-dependent nonequilibrium flow such as pulsed blood flow, the kinematic viscosity $\nu =\frac{\eta }{\rho }$ in Eq. 2 becomes important. Essential diffusive processes such as passive and facilitated transport across cellular and intercellular membranes are set by *D*.

Ascertaining the values of η_0_, ν_0_, and *D*_0_ requires an input from biochemistry and biology. Here, I pose the question of what η_0_, ν_0_, and *D*_0_ are for such an interdisciplinary research. Other questions are: Which life processes are most sensitive to changes of η, ν, and *D* and are disabled first at each stage of life development? How does a function slow down at higher η and lower *D* and what is the nature of a living-to-nonliving transition at high η or low *D* at each stage? What is the effect on other dependent processes? Can we envisage other life forms where these effects are different? Regardless of implications for fundamental constants, these questions are probably interesting in life sciences on their own.

Regardless of what η_0_, ν_0_, and *D*_0_ are, interesting qualitative insights emerge. Combining Eq. 8 with Eqs. 4, 7, and 1 gives

$$\begin{array}{c} \frac{e^{6}}{\hbar^{5}}\sqrt{m_{p}m_{e}^{5}}<\eta_{0}\\ \frac{\hbar^{5}}{e^{6}}\frac{1}{\sqrt{m_{p}m_{e}^{5}}}>D_{0}\\ \frac{\hbar }{\sqrt{m_{e}m_{p}}}<\nu_{0} \end{array}$$ (9)

where I skipped numerical factors unimportant to establishing the range of variation of each constant in response to the biofriendly range of η_0_, *D*_0_, and ν_0_.

Inequalities (Eq. 9) show how each constant is constrained, provided other constants do not vary

$$\begin{array}{c} \max\left(\frac{1}{\eta_{0}^{\frac{1}{5}}},D_{0}^{\frac{1}{5}}\right)<\hbar <\nu_{0}\\ \frac{1}{\nu_{0}^{2}}<m_{e}<\min\left(\eta_{0}^{\frac{2}{5}},\frac{1}{D_{0}^{\frac{2}{5}}}\right)\\ \frac{1}{\nu_{0}^{2}}<m_{p}<\min\left(\eta_{0}^{2},\frac{1}{D_{0}^{2}}\right)\\ e<\min\left(\eta_{0}^{\frac{1}{6}},\frac{1}{D_{0}^{\frac{1}{6}}}\right) \end{array}$$ (10)

In Eq. 10, I dropped the conversion factors that can be reinstated using previous equations, for Eq. 10 serves to show a trend. As mentioned earlier, the conditions for η_min_ and *D*_max_ are independent because they come from different processes. Hence, I used the maximum for the constraint on ℏ and the minimum for constraints on *m_e_*, *m_p_*, and *e* in Eq. 9, so that the range (Eq. 10) reflects the mechanism that disables a life process first.

An interesting observation from Eq. 10 is that biofriendly constraints on η, *D*, and ν imply a biofriendly window for ℏ, *m_e_*, and *m_p_*. This is because η_min_ and ν_min_ depend on ℏ, *m_e_*, and *m_p_* in Eq. 9 differently.

I have considered viscosity getting too high and bio-unfriendly due to different fundamental constants increasing the lower viscosity bound. We could also consider changing fundamental constants to reduce viscosity and its lower bound. If viscosity becomes too low and flow and diffusion get too fast, then accumulation of chemicals in cells and organisms can become too large for healthy functions. However, healthy viscosity and diffusion can be recovered by finding different external conditions serving to increase viscosity (see Fig. 1) and decrease diffusion back to their healthy levels if needed. Hence, decreasing the lower viscosity bound is not as arresting for life as increasing the lower bound.

### Fundamental velocity gradient

To complete the discussion of the role of fundamental constants in life processes involving motion and flow, I derive the fundamental velocity gradient that can be set up in biochemical machines (molecular, cellular, intercellular, or other). These machines play a vital role in sustaining cells and life. Let us consider a machine creating an external force acting to move the liquid in or between cells. There is a limit to how efficient these machines are because they are powered by chemical energy, the energy of chemical bonds with a characteristic scale set by Eq. 5. Let us consider a liquid flowing with constant speed *u* in direction *x* in a volume *V*. The viscous stress is $\sigma_{x}=\eta \frac{\partial u}{\partial y}$, where *y* is perpendicular to *x* and $v=\frac{yu}{l}$ in a simple planar geometry, where *l* is distance between planes (*25*). The viscous force is $f_{x}=\eta \frac{u}{l}S$, where *S* is the area across our volume. The work to move the liquid distance *x* is then $A=\eta \frac{u}{l}Sx=\eta \frac{u}{l}V$. The energy to do this work comes from released chemical, cohesive, energy *E* (e.g., in the Krebs cycle in the metabolic flux), so we write $\frac{E}{V}=\eta \frac{u}{l}$. *E* can be written as *NE*_0_, where *N* is the number of energy-releasing centers in a chemical network and *E*_0_ is the cohesive energy in one bond, whose order of magnitude is given by *E*_R_ in Eq. 5. *V* can be written as *NV*_0_, where *V*_0_ is the elementary volume approximately given by $a_{B}^{3}$. This gives

$$\eta \frac{u}{l}=C\frac{E_{R}}{a_{B}^{3}}$$ (11)

where the coefficient *C* absorbs different factors such as the density of energy-releasing centers, their energy and size in relation to *E*_R_ and *a*_B_, the geometry of the molecular or cellular machine, and so on. *C* is expected to be *C* ≪ 1 because *E*_R_ is larger than a typical energy released in one event in the metabolic flux and *a*_B_ is smaller than a typical size of the energy-releasing center.

I now recall the lower bound for viscosity discussed earlier, η_min_ < η. Combining this inequality with Eq. 11 gives

$$\eta_{\min}\frac{u}{l}<C\frac{E_{R}}{a_{B}^{3}}$$ (12)

Writing η*_m_* = ν*_m_*ρ, $\rho =\frac{m}{a_{B}^{3}}$, *m* = *Am_p_* and using Eqs. 1 and 5 as before, I find

$$\begin{array}{cc} & \frac{u}{l}<{\left(\frac{u}{l}\right)}_{\max}\\ & {\left(\frac{u}{l}\right)}_{\max}=\frac{C}{8\pi \sqrt{A}\varepsilon_{0}^{2}}\frac{m_{e}e^{4}}{\hbar^{3}}{\left(\frac{m_{e}}{m_{p}}\right)}^{\frac{1}{2}} \end{array}$$ (13)

Equation 13 gives the upper bound for the velocity gradient that can be set up by a biochemical machine powered by the chemical bond energy in terms of fundamental physical constants.

Using Eq. 13, I introduce the fundamental velocity gradient set by fundamental constants as

$${\left(\frac{u}{l}\right)}_{f}\propto \frac{m_{e}e^{4}}{\hbar^{3}}{\left(\frac{m_{e}}{m_{p}}\right)}^{\frac{1}{2}}$$ (14)

with the dimensionality inverse of τ_min_ in Eq. 6.

### Variability at fixed α and β

I have considered how bounds on viscosity, flow, diffusion, and velocity gradient can change in response to varying fundamental constants. This variation should be constrained because it should avoid the range where the production of lower-level structure, such as atoms, is disabled. In particular, the fine-structure constant $\alpha =\frac{e^{2}}{\hbar c}$ and the proton-to-electron mass ratio $\beta =\frac{m_{p}}{m_{e}}$ are considered finely tuned in order for heavy nuclei to be produced in stars (*1*, *8*, *9*). Hence, I fix α and β to reflect this tuning and ask how this affects viscosity, diffusion, and flow. One way to write η_min_ in Eq. 4, *D*_max_ in Eq. 7, ν_min_ in Eq. 1, and ${\left(\frac{u}{l}\right)}_{f}$ in Eq. 14 in terms of α and β is

$$\begin{array}{cc} & \eta_{\min}\propto {\left(\frac{e^{2}}{\hbar c}\right)}^{3}\sqrt{\frac{m_{p}}{m_{e}}}{\left(\frac{m_{e}c}{\hbar }\right)}^{3}\hbar \\ & D_{\max}\propto \frac{1}{{\left(\frac{e^{2}}{\hbar c}\right)}^{3}\sqrt{\frac{m_{p}}{m_{e}}}}{\left(\frac{\hbar }{m_{e}c}\right)}^{3}\frac{1}{\hbar }\\ & \nu_{\min}\propto \frac{1}{\frac{e^{2}}{\hbar c}\sqrt{\frac{m_{p}}{m_{e}}}}\frac{e^{2}}{m_{e}c}\\ & {\left(\frac{u}{l}\right)}_{f}\propto \frac{{\left(\frac{e^{2}}{\hbar c}\right)}^{2}}{\sqrt{\frac{m_{p}}{m_{e}}}}\frac{m_{e}c^{2}}{\hbar } \end{array}$$ (15)

I note that bounds (Eq. 15) derived in nonrelativistic condensed matter physics are not expressible in terms of α only, as is often the case for other bounds and properties in relativistic high-energy physics (*1*, *2*, *8*), but depend on other fundamental constants too.

Fixing α and β still leaves many ways of varying η_min_, *D*_max_, and ν_min_. For example, any change of ℏ, *m_e_*, or *c* in the factor $\frac{m_{e}^{3}c^{3}}{\hbar^{2}}$ in η_min_ and *D*_max_ changes η_min_ and *D*_max_, but this change can always be compensated by changing other constants in α and β to keep α and β intact. This can be done in many ways: Changing *m_e_* can be compensated by *m_p_* to keep β intact, changing ℏ in $\frac{m_{e}^{3}c^{3}}{\hbar^{2}}$ can be compensated by *e* to keep $\alpha =\frac{e^{2}}{\hbar c}$ intact, and so on. Similarly, changing *m_e_* in the factor $\frac{e^{2}}{m_{e}c}$ in ν_min_ changes ν_min_ but can be compensated by *m_p_* to keep β intact or changing *e* in the factor $\frac{e^{2}}{m_{e}c}$ changes ν_min_ but can be compensated by the change of ℏ in α and so on. The fundamental gradient can also be varied in ways that keep α and β intact.

We therefore see that a universe with fundamental constants different from ours can produce heavy elements in stars but have a planet where all liquids have very high viscosity due to large η_min_ in Eq. 15, for example, that of tar or higher and where observers may not emerge. This can be achieved, for example, by increasing *m_e_* and/or decreasing ℏ while keeping α and β constant in Eq. 15 as discussed above. To reduce this high life-disabling η_min_ to its current biofriendly value, we need to dial the fundamental constants back to their current values so that the bounds (Eq. 15) become biofriendly. Hence, we need to tune the same fundamental constants setting α and β (ℏ, *e*, *c*, *m_e_*, *m_p_*) that, importantly, involves tuning, which is additional and different to tuning involved in fixing α and β. This additional tuning due to biofriendly viscosity is not needed for the generation of heavy nuclei and is therefore redundant for heavy nuclei. This redundancy involves vast, up to 15 orders of magnitude, differences between the two processes (generating heavy nuclei and biofriendly viscosity and diffusion in living organisms) in terms of size and similarly large differences in terms of energy.

The above redundancy applies if tight constraints on α are relaxed (*6*). In this more general case, the constraints on the fundamental constants from biofriendly viscosity and diffusion in Eq. 15 are still additional and different from those imposed by the production of heavy nuclei.

These observations bear a relation to questions asked previously: Can we understand the values of fundamental constants on the basis of a theory more fundamental than we currently have (the standard model) (*1*, *2*, *8*–*10*)? How were these constants tuned (*8*, *9*)? One possibility is that fundamental constants were tuned (attained their currently observed values) once. As mentioned earlier, this would involve redundancy.

If redundancy is to be avoided, then we can conjecture that multiple independent tunings were involved. This includes tuning fundamental constants to produce heavy nuclei and additional tunings needed for other observed sustainable structures to emerge. This conjecture of multiple tuning suggests a similarity to biological evolution where functionally similar traits, such as the different optical nerve connections in humans and octopi, were acquired independently. If the analogy is between acquiring a new trait and one act of tuning fundamental constants leading to a new set of these constants, then an organism as a system with multiple separately acquired traits is analogous to the set of observed fundamental constants produced as a result of multiple tunings. The evolutionary mechanism changes the focus of discussion of fundamental constants and their values: Currently, observed constants can be considered analogous to, for example, a set of traits acquired by the human eye as the result of past evolutionary changes. These traits were helpful and stayed, but the question as to the number of traits in this set is not viewed as meaningful. Nor is this set most optimal (a human eye is less optimized as compared to the octopus eye), as is the case with our universe, which could have been more habitable were some fundamental constants different (*6*). An analogy with physics would imply that the observed fundamental constants are the result of nature arriving at sustainable physical structures, but the values of these constants may not need to be derived in a more fundamental theory as considered previously (*1*, *2*, *8*–*10*).

A useful example of such an emergence of sustainable structures in biochemistry are the DNA blocks forming in protocells as a result of positive feedback in the metabolic flux (*16*). This positive feedback not only is a general idea but is also based on specific biochemical reactions: The core metabolism central to life probably started when first catalysts sped up helpful aspects of the metabolic flux in protocells, enabling the conversion of H_2_ and CO_2_ into the fabric of new protocells. The first nucleotides, followed by RNA and DNA, then emerged inside these replicating protocells through the positive feedback: Protocells with more beneficial chemicals replicated better and passed these chemicals to their daughter cells. This led to the insight that “meaning emerged with function”: The DNA, the mathematical structure, emerged in the process of helping protocells get better at copying themselves. This enabled protocells to proliferate and hence sustain themselves (*16*). I discuss this in more detail elsewhere.

In summary, I showed how condensed matter physics and liquid physics, particularly, provides insights into fundamental constants in addition to those discussed in high-energy physics. The fundamental constants have a window constrained by biofriendly viscosity and diffusion. Ascertaining this window quantitatively invites another interdisciplinary input from life sciences and raises new questions such as the living-to-nonliving transition as a function of viscosity and diffusion. Once ascertained, we can compare the biofriendly window with constraints on fundamental constants from high-energy physics (*1*–*9*). We saw that bounds on viscosity, diffusion, and the fundamental velocity gradient in a biochemical machine can all be varied with no implication for the production of heavy nuclei.
